# Effects of Protection and Sediment Stress on Coral Reefs in Saint Lucia

**DOI:** 10.1371/journal.pone.0146855

**Published:** 2016-02-04

**Authors:** Chantale Bégin, Christiane K. Schelten, Maggy M. Nugues, Julie Hawkins, Callum Roberts, Isabelle M. Côté

**Affiliations:** 1 Department of Biological Sciences, Simon Fraser University, Burnaby, BC, V5A 1S6, Canada; 2 GEOMAR, Helmholtz Centre for Ocean Research Kiel, Düsternbrooker Weg 20, 24105, Kiel, Germany; 3 Laboratoire d’Excellence ‘CORAIL’ and USR 3278 CRIOBE EPHE-CNRS-UPVD, 58 Av. Paul Alduy, 66860 Perpignan Cedex, France; 4 Environment Department, University of York, Heslington, York, YO10 5DD, United Kingdom; Université du Québec à Rimouski, CANADA

## Abstract

The extent to which Marine Protected Areas (MPAs) benefit corals is contentious. On one hand, MPAs could enhance coral growth and survival through increases in herbivory within their borders; on the other, they are unlikely to prevent disturbances, such as terrestrial runoff, that originate outside their boundaries. We examined the effect of spatial protection and terrestrial sediment on the benthic composition of coral reefs in Saint Lucia. In 2011 (10 to 16 years after MPAs were created), we resurveyed 21 reefs that had been surveyed in 2001 and analyzed current benthic assemblages as well as changes in benthic cover over that decade in relation to protection status, terrestrial sediment influence (measured as the proportion of terrigenous material in reef-associated sediment) and depth. The cover of all benthic biotic components has changed significantly over the decade, including a decline in coral and increase in macroalgae. Protection status was not a significant predictor of either current benthic composition or changes in composition, but current cover and change in cover of several components were related to terrigenous content of sediment deposited recently. Sites with a higher proportion of terrigenous sediment had lower current coral cover, higher macroalgal cover and greater coral declines. Our results suggest that terrestrial sediment is an important factor in the recent degradation of coral reefs in Saint Lucia and that the current MPA network should be complemented by measures to reduce runoff from land.

## Introduction

In the past few decades, multiple threats have contributed to large declines in coral cover worldwide [1]. This phenomenon has been especially marked in the Caribbean [2], where coral decline on many reefs has been associated with an increase in macroalgal cover [3]. The factors driving this shift include direct impacts on corals from hurricanes, disease, bleaching and runoff, as well as the indirect impacts of the release of top-down control of macroalgae, arising from overfishing of herbivorous fish and mass mortality of the grazing sea urchin *Diadema antillarum* [4, 5]. A high cover of macroalgae reduces the space available for coral recruitment as well as the relative herbivory pressure per unit area, which may result in a positive feedback loop that helps maintain macroalgal dominance [6]. Moreover, corals in contact with macroalgae can exhibit lower fecundity [7], reduced growth [8] and increased mortality [9, 10]. The stability of the macroalgal-rich state has been debated [11], yet it is clear that a higher biomass of herbivores, which exert a stronger control on macroalgae, is beneficial to corals [12–14]. In this context, marine protected areas (MPAs), within which all extractive activities are banned, should be expected to have positive effects on corals through increases in herbivorous fish populations within their boundaries [15, 16].

Regardless of whether they are protected or not, corals remain vulnerable to threats, such as runoff, storms, disease, increasing water temperatures and ocean acidification, which originate outside reserves [17]. While the latter four threats are virtually impossible to manage directly, runoff may be more easily controlled. In areas experiencing high sedimentation rates owing to land-use changes, the benefits to reef health of reducing runoff may be considerable given that sediment has been shown to decrease light available for photosynthesis, inhibit recruitment, reduce growth and cause stress and mortality to a wide variety of corals [18–22]. Controlling runoff, particularly upland of marine protected areas, may be a sound management strategy to enhance coral cover, or at least reduce its rate of loss.

In this study, we examined the effects of marine protection and terrestrial sediment on the benthic composition of coral reefs. We focused on the coral reefs of Saint Lucia, a small volcanic island in the eastern Caribbean, which is at high risk of accelerated erosion owing to its small, steep watersheds which experience high precipitation rates [23, 24]. Indeed, sedimentation accumulation rates on reefs in Saint Lucia are high compared to other reefs in the region (e.g., 1.5–9 times higher than in Saint John [US Virgin Islands] and Puerto Rico) and have increased by at least 60% since ~1950 [25–27]. A series of small no-take MPAs, known as the Soufriere Marine Management Area (SMMA), was created in 1995 on the west coast of the island. These MPAs have had a high level of compliance [28, 29], and within six years of establishment, total biomass of fishes had quadrupled inside the reserves and tripled outside the reserves, with the greatest increase observed for herbivores [30]. Greater biomass of herbivorous fish inside the SMMA compared to adjacent sites was observed again in early 2014 (R. Steneck, personal communication). However, the initial benefits of the SMMA did not extend to coral, which decreased in cover by 35–46% over this 6-year period, owing to disease (in 1997), bleaching (in 1998), hurricane damage (in 1999), as well as chronic sedimentation stress from the Soufriere River [31–34]. In 2001, another no-take MPA–the Canaries-Anse-La-Raye Marine Management Area (CAMMA), was created, 10 km north of the SMMA. The longer-term effect (> 6 years) of the SMMA and the CAMMA on coral has not yet been documented, and is the focus on this paper.

In this paper we specifically ask whether (1) current (i.e., 2011) benthic composition on Saint Lucian reefs, and (2) changes in the cover of major benthic groups over the last decade, vary in relation to protection and terrestrial influence. To answer these questions, we revisited in 2011 multiple sites located in and out of MPAs, which were surveyed in 2001 [33]. To evaluate terrestrial sediment stress, we sampled surface reef sediment and measured the proportion of terrigenous (non-calcareous) material in this sediment. Surface sediment composition has been shown to reflect input of terrestrial sediment in a variety of settings [35–38], including coral reefs in the eastern Caribbean [39].

## Methods

### Study area

**Fig 1:**
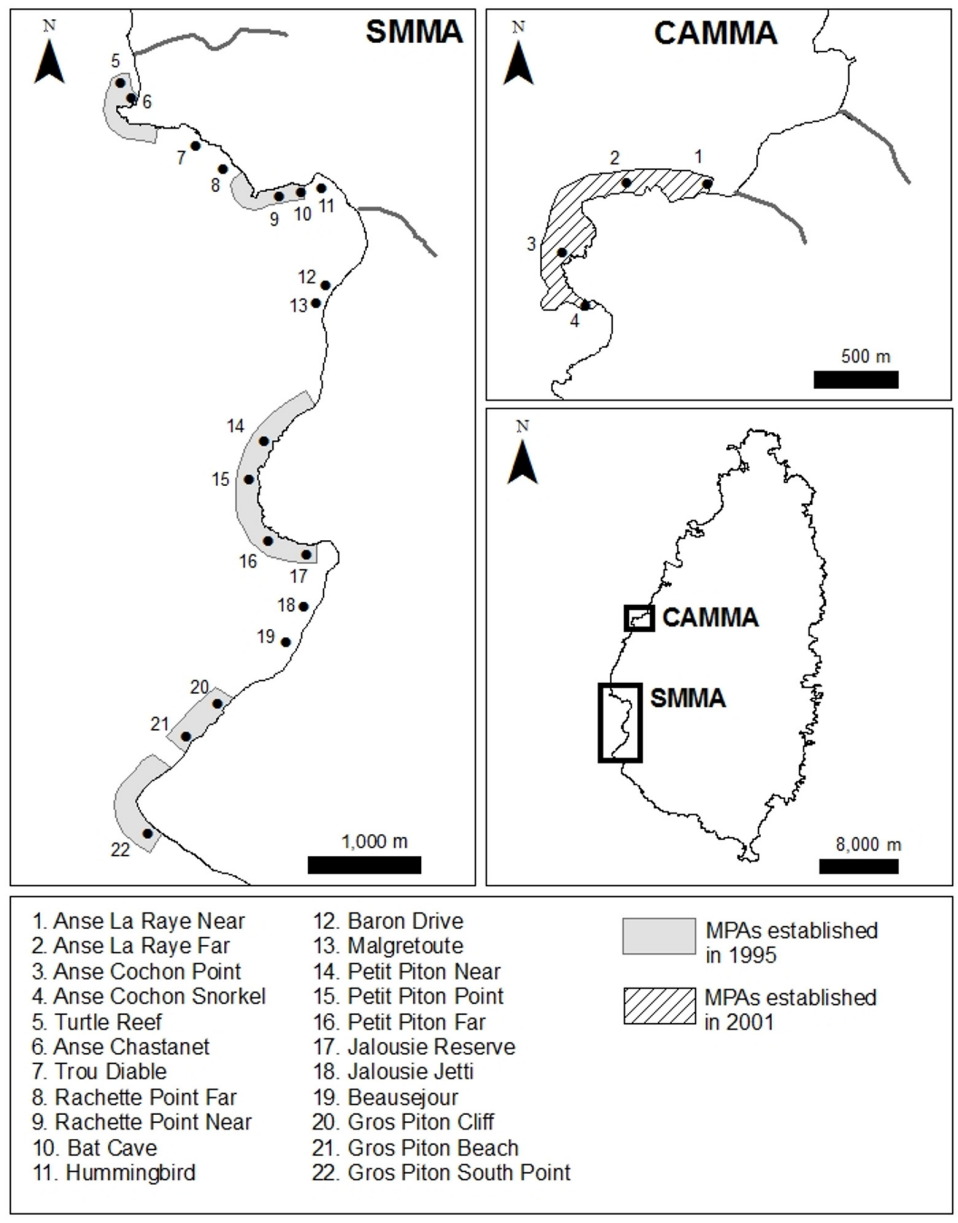
Locations of sampling sites in Saint Lucia surveyed in 2011. All but one site (Site 16) were also surveyed in 2001. Grey areas represent the no-take marine protected areas of the Soufriere Marine Management Area (SMMA), and the hashed area, that of the Canaries-Anse-La-Raye Marine Management Area (CAMMA). The location of the two management areas is shown on the middle-right panel.

Coral reef surveys and sediment sampling were carried out on the west coast of Saint Lucia, in the eastern Caribbean (Fig 1; Table 1). Saint Lucia has a steep terrain and depth increases quickly with distance from shore. Fringing reefs are the typical reef formation around the island, and the only type of reef we surveyed. Fifteen of the survey sites were located inside effective marine protected areas, either within the Soufriere Marine Management Area (SMMA) or the Canaries-Anse-La-Raye Marine Management Area (CAMMA). The Saint Lucia Department of Fisheries provided a research permit.

**Table 1:** Coordinates of survey sites on the west coast of Saint Lucia.

Site	Latitude	Longitude
1. Anse La Raye Near	N13°56.053'	W61°03.148'
2. Anse La Raye Far	N13°56.060'	W61°03.415'
3. Anse Cochon Point	N13°55.828'	W61°03.620'
4. Anse Cochon Snorkel	N13°55.667'	W61°03.550'
5. Turtle Reef	N13°51.881'	W61°04.824'
6. Anse Chastanet	N13°51.814'	W61°04.775'
7. Trou Diable	N13°51.589'	W61°04.438'
8. Rachette Point Far	N13°51.469'	W61°04.321'
9. Rachette Point Near	N13°51.336'	W61°04.051'
10. Bat Cave	N13°51.378'	W61°03.933'
11. Hummingbird	N13°51.416'	W61°03.858'
12. Baron Drive	N13°50.907'	W61°03.820'
13. Malgretoute	N13°50.825'	W61°03.844'
14. Petit Piton Near	N13°50.149'	W61°04.103'
15. Petite Piton Point	N13°49.709'	W61°04.080'
16. Petit Piton Far	N13°49.973'	W61°04.177'
17. Jalousie Reserve	N13°49.647'	W61°03.913'
18. Jalousie Jetti	N13°49.351'	W61°03.900'
19. Beausejour	N13°49.193'	W61°04.018'
20. Gros Piton Cliff	N13°48.884'	W61°04.346'
21. Gros Piton Beach	N13°48.729'	W61°04.503'
22. Gros Piton South Point	N13°48.277'	W61°04.703'

### Early surveys of benthic cover (2001)

Benthic cover at 27 sites along a 16-km stretch of coast was estimated in August and September 2001 using 1-m^2^ quadrats (N = 14–18 per depth), placed at computer-generated, random intervals (1–5 m apart) along a transect at each of two depths, 5 and 15 m, for a total of 28–36 quadrats at each site [33]. Divers estimated visually the percent cover (%) of coral (all species combined), sponges, macroalgae (thick and/or leathery, > 1 cm in height), turf algae (diminutive filaments, < 1 cm in height), crustose coralline algae and filamentous cyanobacteria [33].

### Recent surveys of benthic composition (2011)

In May and June 2011, benthic composition was re-assessed at 21 of the sites surveyed in 2001 and at one additional site (Site 16; Fig 1). Benthic surveys were conducted at 5 and 15 m depth at 18 of the sites, and at only one depth at four sites (5 m only: sites 1 and 6; 15 m only: sites 5 and 19; Fig 1) where reef development was minimal or absent at the other depth. At each site, scuba divers deployed three 30 m transects at each depth haphazardly on the reef, parallel to shore. Transects were at least 10 m apart. Every two meters on each transect, four contiguous photographs were taken from a distance of 0.75 m above the reef. Each photo frame covered 0.41 m x 0.61 m. The four photographs together (totaling 0.82 m x 1.22 m) formed a 1-m^2^ quadrat centered on the transect; there was a total of 15 such quadrats per transect (90 quadrats at each site). To match the method used by Schelten [33], we estimated percent cover of benthic organisms visually on the photographs, using the same benthic types: hard coral, macroalgae, turf algae, sponges, crustose coralline algae, and filamentous cyanobacteria. Transects were the replicate unit for the analysis of benthic cover in 2011, whereas quadrats were used as the replicate unit in the comparisons between 2001 and 2011 since no transects were used in the 2001 surveys.

### Sediment composition

Three replicate samples of sediment (~ 100 ml) per depth were collected with Whirl-pak® bags at each site in 2011 from the surface layer of sediment (to a substrate depth of 5 cm) in a soft-bottom area immediately adjacent (maximum 5 m away) to the area surveyed by transects. Such fore-reef environments tend to have better preservation of sediment compared to back reefs or channels because of lower rates of transport and biogenic re-working [40].

In the laboratory, each sample was rinsed twice with distilled water to remove salts, decanted, and dried at room temperature. Each dry sample was separated into two subsamples by coning [41], one for archiving and the other for composition analysis. Samples were weighed and then treated with a 10% hydrochloric acid solution to dissolve carbonates. Following four rinses with deionized water, samples were dried again and reweighed. The remaining sediments can be assumed to be terrigenous [27, 42]. The proportion of terrigenous sediment was calculated by dividing the weight of the terrigenous fraction by the weight of the subsample prior to treatment with hydrochloric acid. Organic content was determined by loss on ignition (LOI) after at least 2.5 hours at 550°C [43], and was low (< 5%) in all samples. Organic content was therefore not considered in subsequent analyses.

### Statistical analyses

#### Determinants of current benthic composition

We used mixed-effects models and a correlation structure to evaluate variation in the cover of major benthic groups at the 22 sites surveyed in 2011 in relation to three (fixed) effects: sediment composition (i.e., the proportion of terrigenous sediment), depth (5 vs 15 m) and protection status (protected vs unprotected). We included depth as a fixed effect as it was expected to influence community composition [44, 45] and we were interested in its possible interactions with sediment composition and protection status. We also included region (Anse La Raye vs Soufriere) as a random effect. Site could not be included as a random effect in the mixed-effects models because data on terrigenous content of sediment were only available at the site rather than at the transect level. Therefore a compound symmetry correlation structure was also tested by comparing models with one of two correlation terms, i.e. either site within region or region only. Compound symmetry correlation can be used instead of random effects to account for a lack of independent samples caused by correlations among variables across different scales [46]. The analyses were carried out using models with normal errors and constant variance following arcsine transformation of the dependent variable (arcsin(√ (0.01*x))), which is the best method to analyse percentage cover data [47].

For each dependent variable (percent cover of coral, macroalgae, sponges, turf algae, coralline algae, filamentous cyanobacteria), we followed the protocol for model selection outlined in Zuur et al. [46]. First, the optimum random-effects structure was selected by comparing generalized least square (GLS) models that included no random structure to mixed-effect models with random intercept, using the most complex fixed effects (3-way interaction between the proportion of terrigenous sediment, depth and protection status). Models were compared using Akaike’s Information Criterion (AIC). AIC values represent the trade-off between model fit and model complexity, where the lowest value represents the best trade-off. The model with the lowest AIC value was then used to select the most appropriate correlation structure. Random structure was re-tested using the optimum correlation structure. Random and correlation structures were chosen based on minimum AIC scores using the restricted maximum likelihood (REML) estimation method. Finally, the optimum model (with respect to random and correlation structure) was used to select the fixed structure (terrigenous content, depth and protection status), where terms that were not significant were successively removed until all variables remaining significantly improved model fit. Fixed structure selection was carried out using maximum likelihood (ML). Final models were computed using REML. Homogeneity was assessed by graphical methods [46]. Because we observed no heterogeneity in the residuals, we did not test for model improvements by including variance parameters. All analyses were conducted with the “nlme” package in R (version 2.13.1).

#### Determinants of decadal changes in benthic composition

We calculated the changes in cover of major benthic groups between 2001 and 2011 in relation to protection status and sediment composition. Changes in benthic cover at these sites from 1996 to 2002 have been reported previously [30, 33]. We chose to focus on the decade 2001–2011 to cover a period after the 1998 bleaching event and the passage of Hurricane Lenny in 1999, both of which caused extensive mortality on the reefs of Saint Lucia [33]. Moreover, this time period covers the first decade since the establishment of the CAMMA.

We first evaluated absolute changes in benthic cover of each major group over the last decade using repeated-measures ANOVAs, with sites as the subjects and year as the within-subject factor. Only sites/depths for which data were available in both 2011 and 2001 were included in this analysis. Because quadrats used in reef surveys were not permanent and their exact location changed with each survey, site was used as the unit of repeated measurement in these analyses, with site-level benthic cover obtained by averaging across all quadrats (irrespective of transect for 2011). To account for the higher likelihood of finding spurious significant results in multiple tests, we adjusted p-values using the false discovery rate (FDR) [48–50]. For each of the significance tests we calculated the FDR-adjusted significance threshold (α_FDR_) using the adapted linear step-up procedure [51] and report the q-value (FDR-adjusted P-value) [52]. Repeated-measures ANOVAs were performed with IBM-SPSS Statistics 22.

We then estimated the importance of terrigenous content of sediment, protection status and depth for the annual rate of (relative) change in benthic cover of each major benthic group using mixed-effects models as described above. For this analysis, changes in benthic cover were calculated relative to the original survey (i.e., (100*([Cover_end_−Cover_start_]/Cover_start_)/year), similar to Gardner et al. 2003 [2]) to control for varying initial cover, and expressed as annual rates in terms of percentages. In these mixed-effects models, the only random factor was region. The site effect was not accounted for in a compound symmetry correlation since data were only available at the site level. To test whether the disparity in survey effort between 2001 and 2011 (more quadrats sampled in 2011) had an effect on the results, we reran the analyses using only 18 randomly selected quadrats per depth per site for 2011, to match the 2001 sample size per site. The results were very similar, and therefore the results of the analyses with all 2011 quadrats are reported here.

#### Sediment composition at protected and unprotected sites and across depths

We performed a 2-way ANOVA to compare the proportions of terrigenous sediment in and out of MPAs and at shallow (5 m) and deeper (15 m) depths. The mean value of the three replicate sediment samples for each site/depth was used in the analysis. The dependent variable was arcsine-transformed prior to the analysis.

## Results

### Determinants of current benthic composition

**Fig 2:**
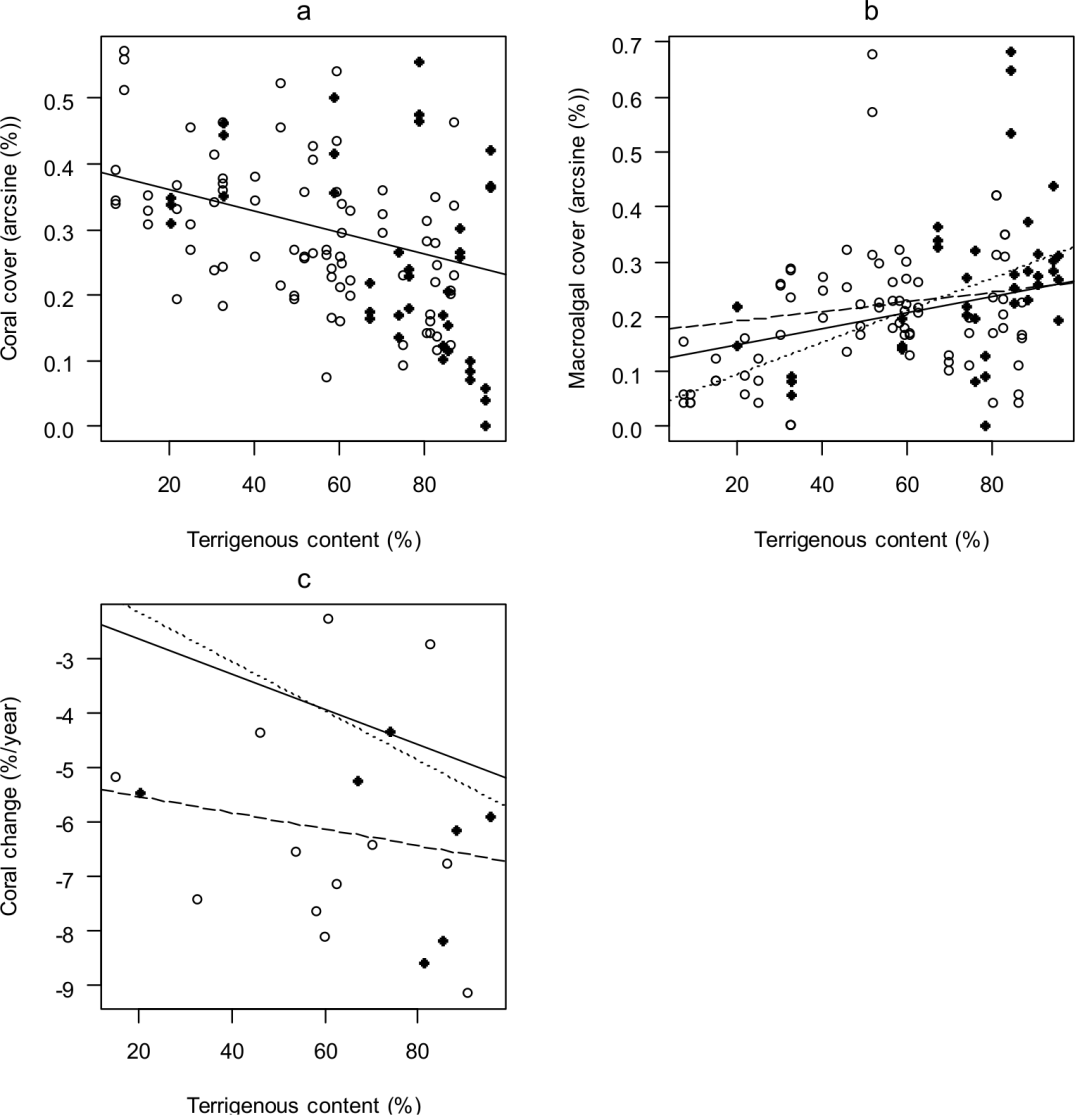
Benthic cover in relation to terrigenous content of sediment adjacent to coral reefs in Saint Lucia, eastern Caribbean. Cover (arcsine %) of (a) coral, (b) macroalgae in 2011, and (c) annual rate of relative change in coral cover from 2001–2011 (see Methods for equation). (a-b): *N* = 60 transects at each depth; (c): *N* = 19 sites at each depth. Solid lines represent regressions including both depths combined: dotted lines represent regressions for sites at 5 m, and long-dashed lines represent regressions for sites at 15 m. Open circles represent sites inside MPAs; filled circles, outside MPAs.

**Fig 3:**
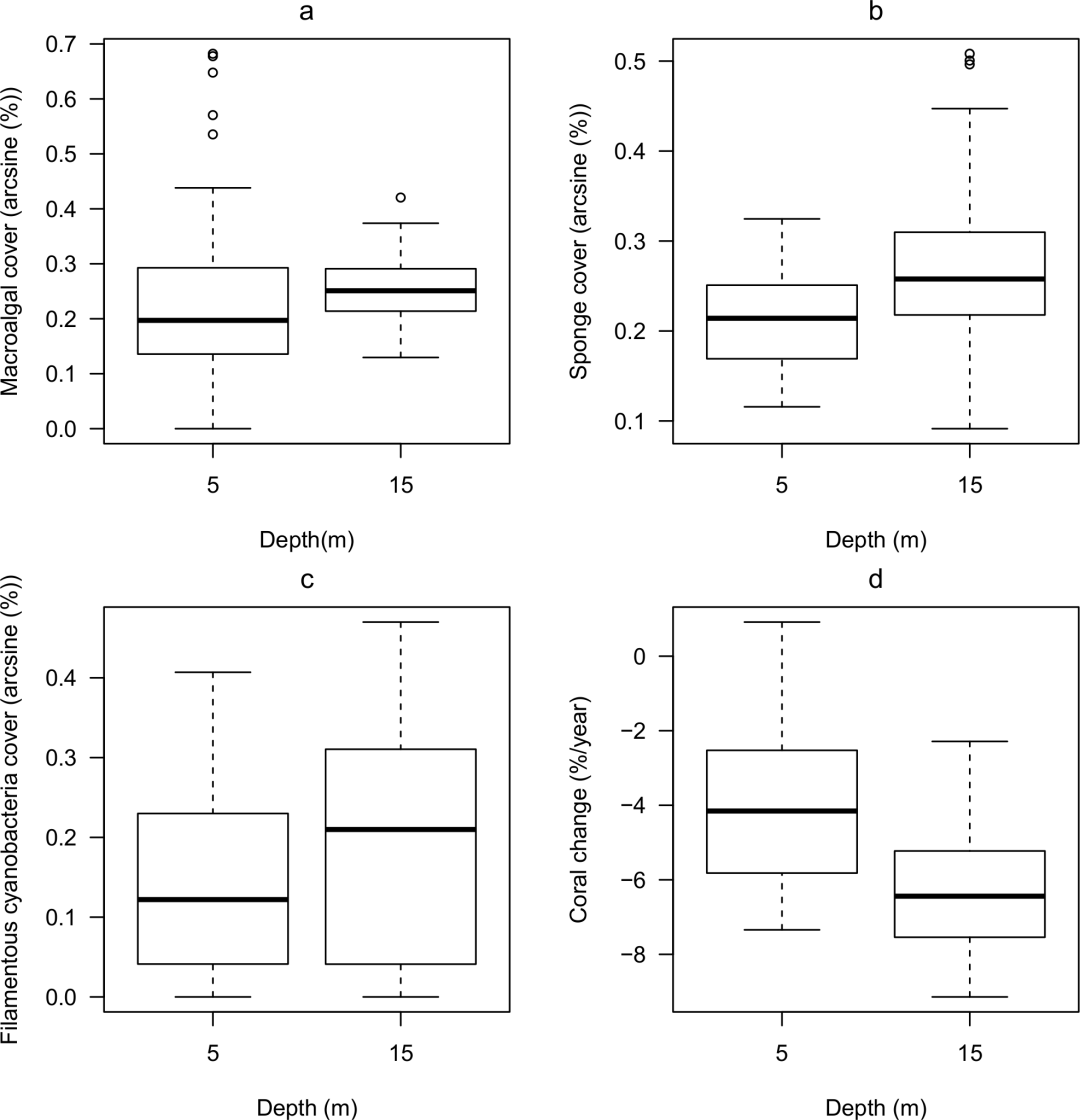
Benthic cover at depths of 5m and 15 m on coral reefs of Saint Lucia, eastern Caribbean. Cover (arcsine %) of (a) macroalgae, (b) sponge and (c) filamentous cyanobacteria in 2011, and (d) annual rate of relative change in coral cover from 2001–2011 (see Methods for equation). (a-c): *N* = 60 transects at each depth; (d): *N* = 19 sites at each depth. Medians are shown by the thick horizontal bars; boxes include the upper and lower quartiles; dashed lines show the range of all values except outliers.

Protection status was not a significant predictor of current benthic cover in any of the models (Table 2). However, there were significant relationships between the cover of four of the six major benthic components considered and either sediment composition or depth or both. In all cases the best model was a generalized least-square with region/site correlation terms (Table 2). Current coral cover was best explained by sediment composition, with higher coral cover when terrigenous content was lower (Fig 2A). Macroalgal cover increased with terrigenous content (Fig 2B) and depth (Fig 3A). There was a significant negative interaction between those factors (Table 2), indicating that the increase in macroalgal cover with terrigenous content was significantly slower at 15 m than at 5 m (Fig 2B). Cover of sponge and filamentous cyanobacteria were both best explained by depth only. Both were more abundant at 15 m than at 5 m (Fig 3B & 3C). The cover of coralline algae and turf algae did not vary with either protection, sediment composition or depth.

**Table 2:** Results of mixed-effects models explaining reef benthic composition in 2011, and annual rate of change in coral cover (2001–2011) in Saint Lucia. Parameter estimates, standard errors (SE), and significance statistics are given. Cover data were arcsine transformed. Annual rate of change in coral cover was calculated relative to initial cover (see Methods). The reference level for the “depth” variable was 5 m; positive estimates for depth therefore reflect a greater response at 15 m than at 5 m.

Dependent variable	Parameter	Estimate	SE	t	P
Coral	Intercept	0.396	0.045	8.12	<0.0001
	% terrigenous	-0.001	0.001	-2.09	0.04
Macroalgae	Intercept	0.068	0.062	1.10	0.27
	% terrigenous	0.002	0.001	2.88	0.005
	Depth	0.242	0.073	3.32	0.001
	% terrigenous*depth	-0.003	0.001	-2.88	0.005
Sponge	Intercept	0.211	0.014	15.35	<0.0001
	Depth	0.064	0.013	4.88	<0.0001
Filamentous cyanobacteria	Intercept	0.141	0.030	4.79	<0.0001
	Depth	0.051	0.014	3.66	<0.0001
Change in coral cover	Intercept	-1.994	0.898	-2.22	0.03
	% terrigenous	-0.032	0.013	-2.43	0.02
	Depth	-2.108	0.665	-3.17	0.003

### Decadal changes in benthic composition

**Fig 4:**
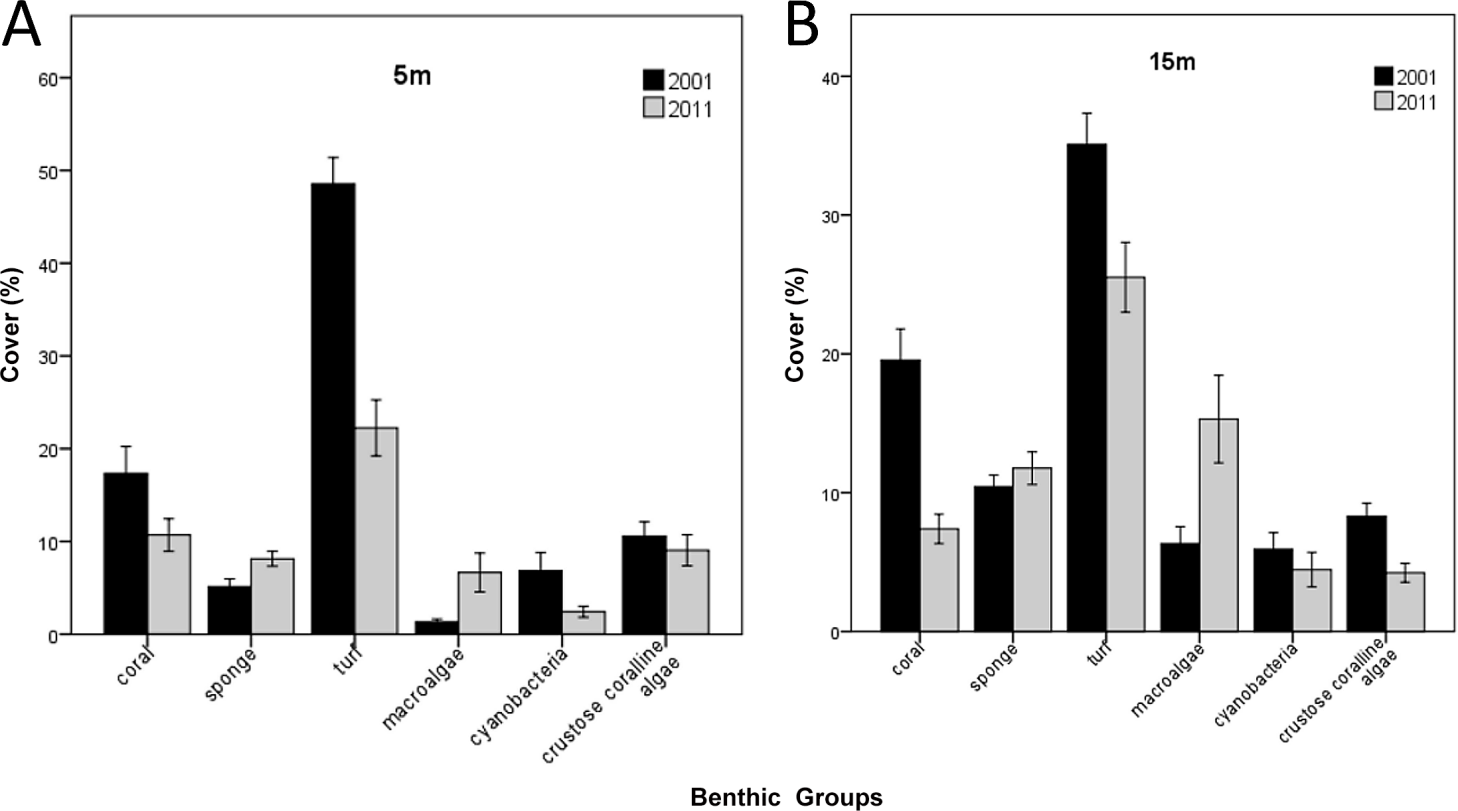
Mean cover of major benthic groups surveyed at depths of 5 m and 15 m, in 2001 and 2011, on coral reefs in Saint Lucia, eastern Caribbean. Bars represent means + 1 standard error. *N* = 19 sites at each depth, for each year.

The patterns of absolute change in cover of benthic components from 2001 to 2011 were similar at 5 m and 15 m depth, although initial cover and magnitude of change varied (Fig 4). There were significant differences between 2001 and 2011 in the cover of all major benthic groups considered, except for crustose coralline algae at 5 m (Table 3) and sponge at 15 m (Table 4). Most notably, mean coral cover decreased by 7–12% (in absolute terms) and turf algae by 10–26%. Macroalgal cover increased by 5–9% over the same decade.

**Table 3:** Results of repeated-measures ANOVAs comparing the cover of major benthic components at 5 m depth on coral reefs of Saint Lucia between 2001 and 2011. Mean change in absolute percent cover for each group is given, with standard error in parentheses. Mean changes do not sum to zero because the cover of sand and rubble was omitted from the analysis. q represents the FDR-adjusted P-value (see Methods). * denotes significant change in cover.

Benthic component	Mean change in absolute cover (%)	F_1,18_	q
Coral	-6.63 (1.9)	12.52	0.004*
Sponge	+2.99 (0.7)	20.76	0.0003*
Turf algae	-26.3 (5.3)	25.96	0.0003*
Macroalgae	+5.3 (2.0)	7.57	0.02*
Cyanobacteria	-4.4 (2.0)	4.96	0.05*
Crustose coralline algae	-1.5 (1.6)	0.90	0.35

**Table 4:** Results of repeated-measures ANOVAs comparing the cover of major benthic components at 15 m depth on coral reefs of Saint Lucia between 2001 and 2011. Mean change in absolute percent cover for each group is given, with standard error in parentheses. Mean changes do not sum to zero because the cover of sand and rubble was omitted from the analysis. q represents the FDR-adjusted P-value. * denotes significant change in cover.

Benthic component	Mean change in absolute cover (%)	F_1,18_	Q
Coral	-12.4 (1.6)	57.99	0.0003*
Sponge	+1.8 (1.5)	0.88	0.36
Turf algae	-9.5 (2.8)	11.25	0.008*
Macroalgae	+8.8 (2.8)	10.06	0.009*
Cyanobacteria	-1.6 (1.1)	1.65	0.03*
Crustose coralline algae	-4.1 (0.8)	24.79	0.0003*

Variation in annual rate of relative change in coral cover over the last decade was best explained by a generalized least-square model, with depth and proportion of terrigenous sediment as fixed factors (Table 2). Protection status was not a significant factor. The annual rate of coral loss was higher at sites that were deeper (Fig 3D) and at sites with a higher proportion of terrigenous sediment (Fig 2C). There was no effect of protection or sediment composition on the rate of change of any other benthic component analyzed.

### Sediment composition at protected and unprotected sites and across depths

The proportion of terrigenous sediment was lower at sites within MPAs (mean ± standard deviation; protected: 52.7 ± 24.4%; unprotected: 71.8 ± 23.3%; F_1,36_ = 6.30; P = 0.02), but similar at each depth (5 m: 55.4 ± 27.1%; 15 m: 63.2 ± 24.0%; F_1,36_ = 0.60; P = 0.45). There was no interaction between protection and depth (F_1,36_ = 0.06; P = 0.81).

## Discussion

This study shows significant shifts in coral reef community composition in Saint Lucia between 2001 and 2011 and suggests strong links between the terrigenous content of reef-associated sediment–a proxy of terrestrial influence on reefs–and both the rate of coral decline and current coral cover. The layer of sediment collected in this study (i.e., the top ~5cm) represents approximately 5 years of accumulation at sites with high sediment load (i.e., 1.34 g cm^-2^ yr^-1^ on Saint Lucian reefs; [53] and even longer at sites with slower accumulation. It therefore reflects the nature of the sediment deposited at a site, and thus the strength of the terrestrial influence, over a substantial portion of the decade we examined. Importantly, protection status appeared to have no impact on benthic assemblages, even though the terrigenous fraction of sediments inside MPAs was lower than that at unprotected sites. The MPAs of Saint Lucia, while successful at protecting fishes [30], have not had a similar effect on corals, which mirrors observations from other Caribbean locations [54, 55].

### Current reef community composition

The cover of coral and macroalgae in 2011 varied significantly with the proportion of terrigenous content in reef sediment. This relationship was negative for coral, which is consistent with the documented detrimental effects of sediment on coral [19, 22, 56–58], and with a large-scale study showing a strong spatial association between low coral cover and high terrestrial influence on Caribbean reefs [39]. On the other hand, there was a positive relationship between the cover of macroalgae and terrigenous content of sediment, at least at the shallower depth (5 m). These results contrast with a previous study conducted throughout the eastern Caribbean, in which macroalgal abundance was negatively correlated with levels of terrigenous sediment [39]. The discrepancy may be caused by differences between the two studies in the dominant algal species. In both cases, the two most abundant taxa were *Lobophora variegata* and *Dictyota* spp. In the present study, the overall mean macroalgal cover was 5.8 ± 7.0% (mean ± standard deviation), with the majority of cover provided by *L*. *variegata* (5.0 ± 9.5%) and only a small proportion by *Dictyota* spp. (0.3 ± 0.6%). By contrast, in the eastern Caribbean study (11 islands between the US Virgin Islands and Grenadines), the mean macroalgal cover was substantially higher (19.3 ± 12.2%), with the cover of *Dictyota* spp. (13.2 ± 10.3%) exceeding greatly that of *L*. *variegata* (2.7 ± 4.8%). *Dictyota* cover varies seasonally and usually peaks in the summer [59], but both this study and that of Bégin et al. [39] were carried out in late winter and spring. The contrasting results could be explained if *L*. *variegata* has a higher tolerance to sedimentation than *Dictyota* spp. To our knowledge, the responses of these macroalgae to sediment load and composition have not been directly investigated, and more research is needed to fully understand these results.

The current cover of several benthic components (macroalgae, sponges, filamentous cyanobacteria) varied significantly with depth in a manner consistent with known patterns of depth zonation on coral reefs [44, 45, 60–62]. There was a significant negative interaction between depth and terrigenous sediment in explaining spatial variation in the cover of macroalgae. Macroalgal cover increased more rapidly with increasing terrigenous fraction in sediment at 5 m than at 15 m. This result was driven mostly by high cover of macroalgae at two shallow sites (Anse Chastanet [site 6] and Malgretoute [site 13], with 25.9% and 34% cover, respectively; Fig 1), but it is not clear what promoted macroalgal growth at these two sites.

### Decadal changes in reef community composition

There was a sharp decline in coral cover between 2001 and 2011, which mirrored a similar change recorded between 1995 and 2001[30]. The reduction in coral cover over that earlier period was attributed to sedimentation, disease, bleaching and a hurricane [31, 33]. To explain our results, once again the multiple stressors that have acted on Saint Lucian reefs in the past decade need to be considered. For example, hurricanes Dean in 2007 and Tomas in 2010 generated significant rainfall and storm surge, although they caused limited swell [63, 64], suggesting that they likely impacted corals mainly through increased sedimentation rather than direct breakage. There was also a substantial bleaching event throughout the Caribbean in 2005 [65], although Wilkinson and Souter [66] reported that it caused little coral mortality and no change in coral cover in Saint Lucia. Our finding that decline in coral in the past decade was significantly related to the proportion of terrigenous sediment at a site suggests that terrestrial sediment is a particularly important factor in driving this change.

The cover of all benthic components other than coral also changed significantly at one or both depths between 2001 and 2011, but unlike coral, these changes were not clearly associated with sedimentation. Of particular interest is the increase in macroalgae, which occurred across all sites, regardless of depth, terrestrial influence or protection status. Although the cover of macroalgae remains low (~7–15%) in absolute terms (see also [67]), this increase is of concern as both *Dictyota* spp and *L*. *variegata*, the most common species at our sites, have been shown to inhibit coral growth, increase mortality [9, 68] and reduce coral fecundity [7]. Shifts in benthic dominance towards lower coral cover and increased macroalgal cover have been reported on many other Caribbean reefs [3, 69, 70], including some in remote areas that have little human influence, signaling that these changes are not only or necessarily caused by local anthropogenic factors.

### Implications for coral reef management

Good spatial protection in Saint Lucia between 1995 and 2011 did not affect benthic assemblages in 2011 or changes in the biotic composition of coral reefs in the decade preceding that. While the cessation of fishing is expected to indirectly aid corals through increases in fish herbivory and associated reduction in macroalgal cover [14], increases in coral cover have been reported in very few MPAs [15, 71]. While the creation of MPAs in Saint Lucia led to rapid increases in fish biomass, and in particular of herbivorous fishes between 1995 and 2002 [30], the cover of macroalgae continued to increase both in and out of the reserves between 2001 and 2011, while coral cover steadily declined. These results suggest that factors other than herbivory are controlling variation in coral cover, and that reef management in Saint Lucia which relied largely on MPAs, was not sufficient to successfully conserve corals over this time period. The stakes in losing live coral may eventually be high. Declines in coral are likely to lead to a loss of architectural complexity of reefs [72] as well as in fish diversity and abundance [73, 74]. The deterioration of coral reefs in Saint Lucia also has strong economic and social implications, as 11% of the national GDP comes from reef tourism ([28]; Saint Lucia Central Statistics Office). Moreover, degraded reefs offer a lower level of coastal protection against storms compared to healthy reefs [75].

Our study shows that the influence of terrestrial sediment is one factor that is clearly linked to coral success in Saint Lucia. Sediment accumulation rates on reefs in Saint Lucia are high; rates estimated for ~1980 to 2010 using radioisotope dating of sediment cores near reefs in the Soufriere and Anse La Raye regions were more than twice as high as those recorded in the US Virgin Islands and Puerto Rico [25–27]. More importantly, rates of terrestrial sediment accumulation have increased by at least 60% over the past 50–60 years, largely driven by an increase in the network of unpaved roads in upstream watersheds [25]. Despite the fact that MPAs in Saint Lucia generally experience less terrestrial influence than unprotected sites, a strong effect of sedimentation on past and current coral cover is detectable. Hence, in order to conserve coral in Saint Lucia, effort needs to urgently focus on reducing the problem of sedimentation.

There is growing appreciation for the fact that marine ecosystems cannot be managed in an ecological vacuum, and that a cross-ecosystem perspective might offer the greatest likelihood of success [76, 77]. Reducing sediment loads on the coral reefs of Saint Lucia will require the co-operation of agencies responsible for land and marine jurisdictions. Such initiatives towards integrated planning were recently outlined by the government of Saint Lucia, but the project did not proceed due to a lack of political will, financial support, and backing from key institutions [78]. Similar problems of inadequate legislation, enforcement, and stakeholder support remain challenges for long-term land-sea stewardship in many parts of the world [79]. Despite these difficulties, sediment runoff remains easier to control than many other threats to corals such as bleaching, acidification and hurricanes. Sediment reduction should therefore be a management priority in Saint Lucia and other coral reef nations undergoing rapid land use changes. Along with efforts to control sediment runoff, good enforcement of existing marine reserves should be maintained, to foster a healthy fish community and strong top-down control of macroalgae.
